# Procedural Memory Following Moderate-Severe Traumatic Brain Injury: Group Performance and Individual Differences on the Rotary Pursuit Task

**DOI:** 10.3389/fnhum.2019.00251

**Published:** 2019-07-19

**Authors:** Arianna Rigon, Nathaniel B. Klooster, Samantha Crooks, Melissa C. Duff

**Affiliations:** ^1^Department of Hearing and Speech Sciences, Vanderbilt University Medical Center, Nashville, TN, United States; ^2^Department of Neurology, University of Pennsylvania, Philadelphia, PA, United States; ^3^Kennedy Krieger Institute, Baltimore, MD, United States

**Keywords:** traumatic brain injury, individual differences, assessment, rotary pursuit, memory, procedural

## Abstract

The impact of traumatic brain injury (TBI) on procedural memory has received significantly less attention than declarative memory. Although to date studies on procedural memory have yielded mixed findings, many rehabilitation protocols (e.g., errorless learning) rely on the procedural memory system, and assume that it is relatively intact. The aim of the current study was to determine whether individuals with TBI are impaired on a task of procedural memory as a group, and to examine the presence of individual differences in performance. We administered to a sample of 36 individuals with moderate-severe TBI and 40 healthy comparisons (HCs) the rotary pursuit task, and then examined their rate of learning, as well as their retention of learning. Our analyses revealed that while individuals with TBI spent a significantly shorter amount of time on target as a group, they did not retain significantly less procedural learning, and as a group their rate of learning was not different from HCs. However, there were high individual differences in both groups, indicating that some individuals might not be able to take advantage of treatment methods designed to leverage intact procedural memory system. Future work is needed to better assess and characterize procedural memory in individuals with TBI across a larger battery of tasks in experimental and clinical setting as memory and learning status may predict rehabilitation success.

## Introduction

Traumatic brain injury (TBI) affects almost 2 million people annually in the US alone, and leads to high socio-economic costs for both survivors of TBI and their families and caregivers (Roozenbeek et al., 2012, 2013; Schiller et al., 2012). Memory and learning deficits are a pervasive consequence of TBI (Vakil, 2005) and are the most commonly recognized and treated deficit (Murray et al., 2001). For instance, individuals with TBI can often forget appointments, have trouble remembering new people they meet, or repeat the same type of information several times during a single conversation. Memory problems are of particular consequence as they not only influence an individual’s capacity for independence and their quality of life, but they can also interfere with the ability to benefit from and adhere to a rehabilitation protocol (Skidmore, 2015).

Memory is not a unitary function. Rather, it is instantiated in the brain across multiple functionally and anatomically distinct systems (Squire, 1992; Eichenbaum and Cohen, 2001). Classic taxonomies distinguish between working memory (the ability to temporally hold and manipulate small pieces of information) and long-term memory (the ability to encode, store, and retrieve unlimited amounts of information over indefinite periods of time) (Tulving, 1987, 1992). Long-term memory, in turn, is composed of declarative (episodic) memory (the ability to acquire and recall new facts and events) and non-declarative (procedural) memory (the ability to acquire and use skills, habits, and preferences). These distinct forms of memory are specialized in the type of information they process, the time course over which information is encoded, and the flexibility of information retrieval and use (Eichenbaum and Cohen, 2001). Declarative memory, dependent on the hippocampus and other medial temporal lobe structures, supports our ability to rapidly acquire *relational knowledge* about the world (e.g., vocabulary, facts) and the events of our daily lives (e.g., episodic memory), along with the flexible expression of that knowledge in novel contexts (e.g., generalization). Non-declarative memory is dependent on the tuning and modification of cortical and subcortical (e.g., basal ganglia, cerebellum) processors that supports knowledge that results directly from experience, including skills and habits (e.g., riding a bike). Non-declarative learning is characterized as incremental, inflexible, and not accessible to consciousness (Reber et al., 1996), although consciousness alone does not reliably distinguish memory systems (Hannula and Greene, 2012). Yet, while these memory systems are distinct and specialized, in healthy adults they operate in parallel to support the acquisition and use of a wide range of complex human behaviors (e.g., communication, skill learning, social cognition, creativity) critical for interpersonal relationships, academic, vocational, and recreational pursuits, and independent living (Poldrack et al., 2001; Skidmore, 2015).

A common assertion in the literature is that declarative memory is highly vulnerable to impairment following TBI, whereas non-declarative (procedural) memory is intact, or relatively preserved (Ylvisaker and Feeney, 1998; Vakil, 2005; Skidmore, 2015). It should be noted, however, that procedural memory has received significantly less study than declarative memory (Vakil et al., 1994; Watt et al., 1999; Wright et al., 2014). Moreover, the few studies that have investigated procedural memory in TBI show mixed results.

Vakil and Lev-Ran Galon (2014) found in a sample of 29 individuals with TBI and 29 healthy comparison (HC) participants that on a Mirror Reading task (which measures procedural learning) individuals with TBI showed a preserved rate of learning (Vakil and Lev-Ran Galon, 2014). Other work has employed mirror reading, mazes, and a rotary pursuit (RP) task, and found that procedural memory (but not declarative memory) was intact during the acute and subacute phase of a TBI (Ewert et al., 1989); similar findings have also been reported when procedural memory was examined in a sample of survivors of pediatric TBI (Ward et al., 2002). However, a study on a large sample of TBI examined oculomotor performance and reported impairment in procedural learning tasks that increased with injury severity (Kraus et al., 2010) and additional work found that individuals with TBI significantly underperformed HC participants in tasks of non-declarative sequence learning (Vakil et al., 2001). Overall, findings on procedural memory tasks following TBI appear to be mixed, which is likely to be due to the large individual variability present within populations with TBI, the different types of task employed, and the statistical analyses techniques applied to the data (Vakil, 2005).

That non-declarative memory is spared in TBI and can be leveraged in rehabilitation serves as the foundation of a number of therapy approaches (e.g., errorless learning; positive everyday routines) (Clare and Jones, 2008). These approaches seek to compensate for declarative memory deficits (Skidmore, 2015). However, the problem with the umbrella assertion that declarative memory is impaired in TBI while non-declarative memory is spared is that it may not hold true for the population as a whole. Indeed, the previous work cited above suggests that procedural memory is not uniformly preserved in individuals TBI or on all assessments of procedural memory. If this is the case, then procedural-memory based treatments are only likely to be successful for some patients and might not yield positive results in others. A treatment approach that is designed to leverage a memory system that is not available to a given individual wastes valuable time and healthcare resources. Yet, we should note that procedural memory abilities are rarely assessed in clinical settings. To truly assess the potential and effectiveness of non-declarative memory based treatments for individuals with TBI, and the status of procedural memory in TBI more broadly, further research is needed.

The goal of the current study was to examine performance on a task of procedural memory in a large sample on individuals with moderate-severe TBI and demographically-matched HC participants, to investigate whether (1) as a group, individuals with TBI underperform healthy individuals, and (2) within the sample of individuals with TBI, there are individual differences in procedural memory and learning performance. In particular, we used RP (Ammons, 1955), a task that has been widely employed to assess motor learning and procedural memory in a number of clinical populations with motor and/or procedural and memory dysfunctions, including TBI, Huntington’s disease, and Parkinson’s disease (Harrington et al., 1990; Gabrieli et al., 1997; Haaland et al., 1997). Performance on the RP is spared even in individuals with severe declarative amnesia and Alzheimer’s disease, and thus the task is considered a pure measures of motor-perceptual learning, and has been treated as a gold standard assessment of procedural memory in the literature (Corkin, 1968; Heindel et al., 1989; Finn et al., 2017). Our aims here were twofold: first, we examined the presence of group differences on both rate of learning and retention of motor learning between individuals with TBI and HCs. Second, we investigated the presence of individual differences in rate of learning within the TBI group.

## Materials and Methods

### Participants

Thirty-six individuals with moderate-severe TBI and 40 HC participants were tested. Participants were recruited through The University of Iowa Brain Injury Registry and through The University of Iowa community (Rigon et al., 2016a,b, 2017). Inclusionary criteria for individuals with TBI included a history of moderate-severe TBI and chronic post-injury phase (all participants were > 6 months post-injury). Language deficits were ruled out to ensure that participants were able to understand instruction, and that poor performance on the tasks administered was not due to such deficits. The groups were not significantly different for age [*t*(72.14) = 1.72, *p* = 0.09], education [*t*(69.84) = 1.58, *p* = 0.12], or sex [*X^2^*(1, *N* = 76) = 2.67, *p* > 0.05] (Table 1); results were obtained using Welch *t*-tests.

**TABLE 1:** Participant demographics.

	***N***	**Age *(Mean ± SD)***	**Sex** (Females)**	**Education*(Mean ± SD)***	**Chronicity *(Months, Mean ± SD)***
HC	40	52.88 ± 14.16	23	15.15 ± 2.01	N/A
TBI	36	47.11 ± 14.94	13	14.36 ± 2.31	48.72 ± 58.13
Group differences (*p*)	N/A	0.09	0.1	0.12	N/A

*HC, healthy comparison participants; TBI, traumatic brain injury; *p*, *p*-value; SD, standard deviation; N/A, not applicable.*

Participants had sustained their TBI a minimum of 6 months and a maximum of 307 months before testing (Mean = 48.72, *SD* = 58.13). One participant had sustained two separate TBIs. Causes of injury were falls, motor vehicle accidents, assaults, and non-motor vehicle accidents.

Traumatic brain injury severity was assessed using the Mayo Classification System (Malec et al., 2007). Participants were considered moderate-severe if at least one of the following criterion was met: (1) Glasgow Coma Scale (GCS) < 13 (i.e., moderate or severe according to the GCS), (2) positive acute CT findings or lesions visible on a chronic MRI, (3) loss of consciousness (LOC) > 30 min or post-traumatic amnesia (PTA) > 24 h, and (4) retrograde amnesia > 24 h. Injury-related information was collected using a combination of medical records and a semi-structured interview with participants. Only participants whose motor skills were sufficiently intact to complete the RP Task were included in the study; this was determined by asking the participant to hold the metal wand in contact with the moving target at the slowest speed, and excluding participants who were not able to perform the task (see below for further information about the RP task).

Inclusionary criteria for HC were no self-reported history of head injury or loss of consciousness, no history of neurological, psychiatric or learning disorders.

### Rotary Pursuit Task

Procedural learning and memory were measured using the RP task, and following the protocol described by Heindel et al. (1989). In the RP, participants are instructed to keep a metal wand in contact with a moving target as it moves around a circle. The task comprises two sets of eight trials (Trials 0–7 and 8–15) separated by a break of 30–60 min. The length of the interval was decided based on the protocol established by Heindel et al. (1989), who examined performance on the RP in several clinical populations. Each set is composed of two blocks of four trials, for a total of four blocks. Each trial lasts 20 s, with an 8 s break between trials. Learning is shown by increasing the Time on Target across trials. Before the task begins, the baseline for each participant is individually determined by testing participants at four different speeds (15–60 rpm). The speed at which participants achieve what is closest to 25% time-on-target (i.e., 5 out of 20 s) is chosen as the participant’s baseline, to allow room for learning on subsequent trials.

### Statistical Analysis

#### Group Comparison

Data were first checked for outliers using Cook’s distance. There were no observations with a Cook’s distance greater than four times the mean of their respective group, revealing no influential data points. Our first aim was to examine group differences in performance on the RP. We performed two different types of group comparison analyses, to examine (1) Learning, or whether there were group differences in the rate of change across all the trials of the RP task; and (2) Memory, or whether there were group differences in the retention of acquired skill between block one and block two of the RP. In particular, we were interested in examining whether individuals with TBI retained significantly less learning than HCs after the break interval. The two analyses are discussed below in details.

In the current sample, the length of interval between the first and the second block was not significantly different between the groups [*t*(73.02) = 1.92, *p* = 0.06]. However, the group difference did approach significance (*p* = 0.06), with a longer interval for HCs (38.08 ± 6.06) than for individuals with TBI (35.39 ± 6.11). For this reason, length of interval (interval) was added as a covariate in all group comparison analyses. Baseline speed was not significantly different between the groups [*t*(68.82) = 1.22, *p* = 0.23].

In all analyses dummy coding was used, with HC as the reference group.

##### Rate of learning

Here, we examined group differences in rate of learning across the 16 trials of the RP task using mixed effect modeling. In our analysis, fixed effects included main effects of trial (0 to 15) and group (TBI vs. HC), the interaction between trial and group, a quadratic slope, as well as the interaction between group and a quadratic slope, and interval. The quadratic slope was added after visual inspection of the raw data at the subject level. In other words, we examined the existence of group differences at baseline (i.e., during trial 0) and then group differences in the rate of learning (linear slope) and in the acceleration or deceleration of learning (quadratic slope). We used the *lmer* procedure to fit nested models varying in random effect structures, and then an ANOVA procedure to compare said models. *p-*Values were calculated using lmerTest via the Satterthwaite’s degrees of freedom method. We first included in the random effect structure a random intercept for subject, as well as a random quadratic slope for subject, a random set slope for subject and a random trial slope for subject (model 1); the model did not converge. We then eliminated the random quadratic slope (model 2), and then the set slope (model 3). We then compared the two nested models with a log-likelihood ratio test; the final random effect structure included a subject intercept, a linear slope for subject, and a set by subject slope [χ(2) = 9.07, *p* = 0.03) (see Supplementary Materials).

##### Retention of learning

Here, we examined whether there was a significant group difference in the Time on Target between the last trial of the first block (i.e., trial 7) and the first trial of the second block (i.e., trial 8). These two trials are of particular significance because they are separated by an interval, and thus a group by trial interaction allows us to investigate the presence of a significant group difference in skill retention. In this analysis, fixed effects included main effects of trial (7 to 8) and group, as well as the interaction between trial and group. The random effect structure included a subject intercept.

#### Individual Differences

Our second aim was to examine the presence of individual differences in memory and learning within individuals with TBI. The purpose of this analysis was to determine whether, regardless of how individuals with TBI as a group perform in comparison with HCs, specific individuals with TBI are impaired on the RP. As procedural memory-based treatment strategies are often used in the field of TBI rehabilitations (e.g., errorless learning, positive everyday routines) our goal was to determine the presence of a subgroup of individuals who show a significantly slower rate of learning on the RP following their TBI. To identify the presence of such individuals, two different approaches were employed: for *Rate of learning analysis*, the same model described above were run, this time eliminating group from the factorial design. Subsequently, individual slopes for rate of learning were obtained. For *Retention of learning* analysis, the difference trials 7 and 8 was calculated. Density plots were created. Finally, within the TBI group we used Pearson’s correlations to examine the associations between demographic variables (education, age, and chronicity) and individual scores on *Rate of learning* and *Retention*, as well as the relationship between interval lapsing between trial 7 and 8 and retention of learning between trials 7 and 8.

## Results

### Group Analysis

#### Rate of Learning Analysis

**FIGURE 1:**
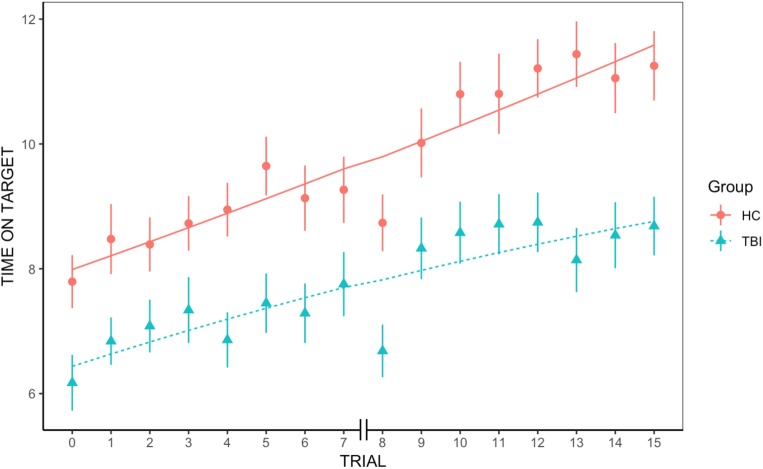
Group differences in rate of learning. We found an overall significant positive effect of trial and a significant effect of group, but no significant group-by-trial interaction, indicating that rate of learning was not significantly different between groups.

We found an overall significant positive effect of trial, indicating that across groups average rate of learning was significantly greater than 0 [*t*(779.3) = 3.45, *p* < 0.001]. We also found an overall significant effect of group on the first trial, with individuals with TBI underperforming HC participants [*t*(91.3) = −2.78, *p* = 0.006]. However, the quadratic slope was non-significant, revealing that the rate of improvement did not significantly accelerate or decelerate across trials [*t*(986) = −0.42, *p* = 0.67]. Similarly, neither the group-by-trial interaction [*t*(779.3) = −0.2, *p* = 0.85] nor the group-by-quadratic slope interaction were significant [*t*(986) = −0.83, *p* = 0.41], indicating that the average rate of learning and the learning acceleration were not different between groups (see Figure 1).

#### Retention of Learning

**FIGURE 2:**
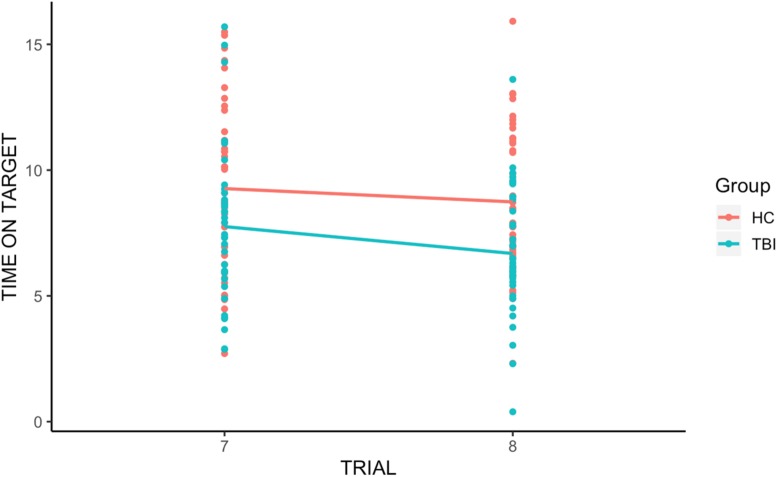
Group differences in retention of learning. We found a significant group effect and a significant trial effect, with Time on Target for trial 8 (the first trial after the break) significantly lower than for trial 7. However, there was no significant group-by-trial interaction, indicating that individuals with TBI and HCs displayed similar retention of procedural learning (memory).

There was a significant group effect [*t*(110.10) = −3.67, *p* < 0.001], with individuals with TBI performing worse than HCs across trials, and a significant trial effect [*t*(66.65) = −2.63, *p* < 0.05], with Time on Target for trial 8 significantly lower than for trial 7. However, there was no significant group-by-trial interaction [*t*(58.75) = 0.32, *p* = 0.75], revealing that the retention of learning was not significantly different between HCs and individuals with TBI (see Figure 2).

### Individual Difference Analysis

#### Rate of Learning

**FIGURE 3:**
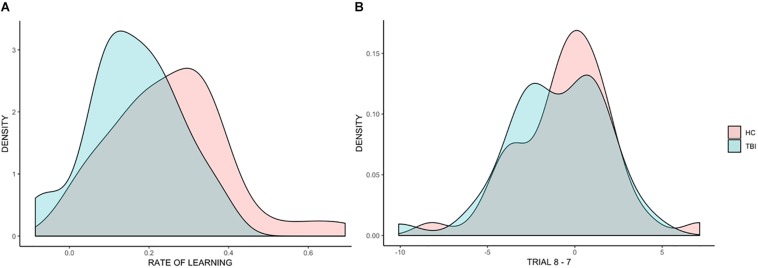
Distributions of individual scores across all analyses. Density plots representing individual differences in the beta coefficients for rate of learning **(A)** and in the difference between trial 8 and trial 7 **(B)**.

Figure 3A shows the distributions of rate of learning for the TBI and HC groups. While there was a high overlap between the distribution within the two groups, with large within group variability, the distribution for the TBI group was shifted to the left compared to the HC group, with more individuals with TBI falling on the tail end of the distribution. This indicates that while statistically not significant, individuals with TBI displayed, on average, lower rates of learning than HC participants.

Within the TBI group we found no significant correlations between rate of learning and education (*p* = 0.76, *r* = −0.05) and rate of learning and chronicity (*p* = 0.54, *r* = −0.11); the correlation between age and rate of learning was negative, and significant (*p* = 0.047, *r* = −0.33).

#### Retention of Learning

Figure 3B shows the distributions of the effect of trial (before the break vs. after the break) for TBI and HC groups, showing a high overlap in the distribution of the two groups.

Within the TBI group we found a significant correlation between retention of learning and education (*p* = 0.01, *r* = −0.41), but no correlations with chronicity (*p* = 0.97, *r* = −0.007), with age (*p* = 0.19, *r* = −0.28) or with duration of interval (*p* = 0.23, *r* = −0.14).

## Discussion

In the current study, we set to examine procedural memory and learning in a sample of 36 individuals with moderate-severe TBI and 40 demographically matched comparison participants using a widely employed motor learning task, the RP. We found that (1) individuals with TBI did show reduced time on target relative to HC participants, although, as a group, their motor learning rate was not significantly different from the HC group, (2) and that acquired procedural memory (i.e., retention of procedural learning) did not significantly differ between groups after a 30–60 min delay. However, there were high individual differences in both groups. Below, we discuss each of our findings.

To our knowledge, this was the first study that took advantage of mixed effect modeling to examine rate of procedural learning following TBI. Here, we examined the slope of learning and the presence of acceleration or deceleration in learning. This analysis revealed that while individuals with TBI generally displayed significantly less time on target across all trials, the rate of learning was not significantly different between groups. This seems to suggest that individuals with TBI retain the ability to learn on new motor tasks and improve with practice. That individuals with TBI spend significantly less time on the target across all trials could be linked to fatigue or reduced processing speed. Overall, these data support and confirm, in a larger sample, the findings of Ewert et al. (1989).

When we examined retention of learning following an interval, we found that, as a group, individuals with TBI and HC participants did not differ (even though for both groups the time on target was lower compared to the time on target pre-interval, as revealed by a significant trial effect). In terms of real-world implications, this reveals that, on average, individuals with TBI who are initially successful on learning tasks of this type can be expected to retain the learning of a new task after a delay (e.g., between one rehabilitation session and another); however, it should be kept in mind that the delay used for the RPT (30–60 min) is much shorter than the time that would pass between one session and another, and investigations that span days, or even weeks, might reveal different results. It is interesting to note that even though there was a dip in time on target for both groups after the inter-set interval (trial 7 and trial 8), by trial 9, participants’ performance returned pre-interval levels. This effect of savings in learning, or relearning, is well-established in the literature (e.g., Cohen and Squire, 1980) and can be observed in skilled learning in TBI as well. We also found individual differences among those with TBI. Reporting findings such as these (i.e., no significant group differences on some measures of learning, with some individuals with TBI who fall at the tail end of the distribution, underperforming the healthy group as well as many other individuals with TBI) is challenging, and care must be taken to not overemphasize one finding over the other. Two points seem clear from the current study. First, the assumption that procedural memory is preserved in individuals with TBI, at least on the task used here, appears to hold at the group level. This suggests that an intervention approach designed to take advantage of preserved procedural memory is likely to be successful for the majority of individuals with TBI. Second, there appears to be a small subset of individuals with TBI for whom procedural memory is not intact. These individuals are likely not to benefit from rehabilitation protocols that leverage intact procedural memory given their deficit in that domain. Recognition of this, albeit a minority of individuals with TBI, is important as we move toward better understanding of the individual factors that can predict or explain treatment responsiveness in the clinic and in clinical research. Correlational analyses revealed a significant correlation between rate of learning and age, indicating that older individuals with TBI tended to show lower rates of learning. However, the correlation was only marginally significant (*p* = 0.047, *r* = −0.33) revealing that other factors are likely to play a role in individual differences. Similarly, retention of learning was significantly and negatively associated with education (individuals with TBI who retained more were also more highly educated). This indicates that while some demographic variables might account for some of the variance in procedural memory following TBI, additional work is necessary to identify the full spectrum of contributing factors. Future research is warranted to develop reliable assessment tools to measure procedural memory in clinical settings and to determine if procedural memory status can predict treatment success in approach that purport to leverage it. Moreover, considering the heterogeneity among individuals with TBI, it will be important to determine the neuropsychological correlates of procedural memory impairment following TBI, to clarify what types of impairment tend to cluster within individuals.

One of the limitations of the current study is that only one task was used to measure procedural memory. A better picture of how procedural learning and memory can be impaired could be obtained by administering a battery of tasks that focus on different aspects of this construct. Moreover, the RP has a strong motor component, and as we only administered the task to individuals who were able to complete it, this might have resulted in the oversampling of individuals with TBI with intact motor skills. As there are other procedural learning tasks that do not rely so heavily on the integrity of motor skills (e.g., mirror reading), it is possible that those studies would be better able to characterize procedural memory more broadly in TBI, and might lead to different results (e.g., more or fewer individuals demonstrating a deficit on a given task or various patterns of spared and impaired performance across a range of procedural memory tasks). Our lab is currently working on this question by testing a larger TBI sample of a battery of procedural memory tasks without and without a motor component. Moreover, it is possible that rate of learning could be different, or impaired, at distinct phases of skill acquisition (early vs. late) based on initial learning success but tasks like the RP are not suited for distinguishing these possibilities. Another limitation, as mentioned above, is the lack of neuropsychological data to further characterize the sample. This, considering the heterogeneity among individuals with TBI, makes it difficult to determine whether the current sample had a neuropsychological profile that is typical of TBI populations, and whether the findings can be generalized to the TBI population as a whole.

Noting these limitations, the current data, together with other reports of disruptions in procedural memory following TBI, suggest that there is a small subset of individuals with TBI for whom procedural memory is not intact. As we stated above, future studies will need to replicate these findings with larger samples to determine the reliability of the effect, as it is possible that group differences in procedural memory might be observable with in larger samples. Given the well-documented heterogeneity in cognitive performance and outcome among individuals with TBI, the current study highlights the importance of combining analyses at both the group and individual level. We suspect that such an approach will move the field away from blanket statements of preserved or impaired ability in TBI and toward more nuanced consideration of performance across individuals and those factors and mechanism that underlie it. We suggest that it is only then that we will truly be able to understand the heterogeneity that is hallmark in TBI and to deliver individualized interventions.

In this vein, future work should determine the reliability of various procedural memory tasks and normative properties to determine their suitability for use in clinical settings. Indeed, we believe that how procedural memory is affected by TBI is worth further study and consideration, even if deficits in procedural memory are the exception following TBI rather than rule. At the level of the individual, the presence of procedural memory impairment, even subtle deficits, could undermine or slow rehabilitative efforts.

## Conclusion

The currently study reveals that, as a group, individuals with TBI do not differ from HC participants on a measure of procedural memory and learning; however, impairment can exist at the individual level. More extensive characterization of memory and learning abilities in individuals with TBI promises to illuminate the observed heterogeneity in cognitive and behavioral outcomes and to guide clinical decision making at the level of the individual.

## Data Availability

The datasets generated for this study are available on request to the corresponding author.

## Ethics Statement

This study was carried out in accordance with the recommendations of the Department of Health and Human Services (DHHS) of The University of Iowa with written informed consent from all subjects. All subjects gave written informed consent in accordance with the Declaration of Helsinki. The protocol was approved by the Institutional Review Board.

## Author Contributions

NK and MD designed the study. NK and SC executed the experiment. AR and MD analyzed the data. AR, MD, and NK wrote the manuscript.

## Conflict of Interest Statement

The authors declare that the research was conducted in the absence of any commercial or financial relationships that could be construed as a potential conflict of interest.
