# Supplementary material for: Procedural Memory Following Moderate-Severe Traumatic Brain Injury: Group Performance and Individual Differences on the Rotary Pursuit Task
Source: Front Hum Neurosci. 2019 Jul 19;13:251. doi: 10.3389/fnhum.2019.00251 (PMC6658892; doi:10.3389/fnhum.2019.00251)
Supplement: Supplementary file 1 [file Data_Sheet_1.docx]

**Supplementary Information**

Models run to determine the random effect structure of the rate of learning analysis.

model1 <- lmer(tot ~ trialn*group + interval + I(trialn^2)*group + (1 + trialn + set + I(trialn^2)|sub), data = RP_data_long)

Did not converge

model2 <- lmer(tot~ trialn*group + interval + I(trialn^2)*group + (1 + trialn + set |sub), data = RP_ long)

Linear mixed model fit by REML. t-tests use Satterthwaite's method ['lmerModLmerTest']

Formula: totarg ~ trialn * group + interval + I(trialn^2) * group + (1 + trialn + block | sub)

Data: RP_ long

REML criterion at convergence: 5245.6

Scaled residuals:

Min 1Q Median 3Q Max

-6.6862 -0.5395 -0.0090 0.5499 6.0594

Random effects:

Groups Name Variance Std.Dev. Corr

sub (Intercept) 4.75332 2.1802

trialn 0.02821 0.1679 0.10

setSecond 1.24592 1.1162 -0.41 -0.35

Residual 3.14277 1.7728

Number of obs: 1216, groups: sub, 76

model3 <- lmer(tot ~ trialn*group + interval + I(trialn^2)*group + (1 + trialn|sub), data = RP_ long)

Linear mixed model fit by REML. t-tests use Satterthwaite's method ['lmerModLmerTest']

Formula: totarg ~ trialn * group + interval + I(trialn^2) * group + (1 + trialn | sub)

Data: RP_ long

REML criterion at convergence: 5254.7

Scaled residuals:

Min 1Q Median 3Q Max

-6.4426 -0.5432 -0.0025 0.5516 5.9329

Random effects:

Groups Name Variance Std.Dev. Corr

sub (Intercept) 5.21231 2.2830

trialn 0.02659 0.1631 -0.18

Residual 3.23088 1.7975

Number of obs: 1216, groups: sub, 76
